# Taking the climate risk out of transplanted and direct seeded rice: Insights from dynamic simulation in Eastern India

**DOI:** 10.1016/j.fcr.2019.05.014

**Published:** 2019-06-01

**Authors:** Balwinder-Singh, A.J. McDonald, Virender Kumar, S.P. Poonia, Amit K. Srivastava, R.K. Malik

**Affiliations:** a International Maize and Wheat Improvement Center (CIMMYT) – India, NASC Complex, DPS Marg, New Delhi, 110012, India; b Soil and Crop Sciences Section, School of Integrative Plant Science, Cornell University, Ithaca, NY, USA; c International Rice Research Institute, DAPO 7777, Metro Manila, Philippines

**Keywords:** *Ex ante* assessment, APSIM, Water stress, Yield stability, Sustainable intensification

## Abstract

Rice productivity in Eastern Indo-Gangetic plains (EIGP) is extremely low, in part due to the prevailing practice of cultivating long-duration transplanted rice under rainfed conditions which leads to water stress and significant yield losses in many seasons. Rice establishment alternatives such as direct seeded rice (DSR) require less water at planting but also are accompanied by climate risks that constrain adoption. For both conventional transplanted and DSR systems, successfully addressing climate-based production risks may provide a strong basis for sustainable rice intensification in EIGP. In this *ex ante* study of rice yield and yield variability, the APSIM cropping system model was used to evaluate the efficacy of risk-reducing management practices in both transplanted and DSR systems. Simulations were conducted with 44 years (1970–2013) of historical weather data from central Bihar, India. Results confirm that the prevailing farmer practice of transplanting long-duration cultivars under rainfed conditions (fTR) often results in delayed transplanting and the use of older seedlings, leading to low (median 1.6 t ha^−1^) and variable (Standard deviation (SD) 2.1 t ha^−1^) rice yields. To improve the fTR system, simulations suggest that adoption of medium-duration hybrid rice (3.2 t ha^−1^), provision of supplemental post-establishment irrigation (3.2 t ha^−1^), or transplanting appropriately aged seedlings (3.4 t ha ^−1^) can double yields as single interventions while, in the case of supplemental irrigation, significantly reducing inter-annual production variability. Additional gains are achievable when interventions are layered: supplemental irrigation paired with medium-duration hybrids increased median rice yields to 4.6 t ha^−1^ with much lower variability (SD 1.0 t ha^−1^). In these improved systems where irrigation is used to transplant the crop, simulations revealed the importance of timely planting: high and stable yields are achievable for long-duration cultivars when transplanting is completed by 2 August with this window of opportunity extending to 16 August for medium-duration hybrids. In rainfed DSR systems, the potential pay-offs from single interventions were even higher with medium-duration hybrids resulting in a median yield of 4.5 t ha^-1^ against 1.8 t ha^−1^ with long-duration cultivars. For irrigated DSR systems, an optimum sowing window of early to mid-June was identified which resulted in higher and more stable yields with lower water requirements. Simulation results suggest several risk-reducing intensification pathways that can be selectively matched to farmer risk preferences and investment capabilities within the target region in EIGP.

## Highlights

• Optimum rice transplanting date in EIGP to achieve high yield with low risk is up to 2 August for long duration variety and is up to 16 August for short duration variety.
• Late transplanting with old seedlings under farmer practice lead to low rice yields due to high drought risk but supplemental irrigation or short duration variety reduced the drought risk.
• Transplanting of appropriate aged seedling at onset of monsoon yields 1.8 t ha^−1^ as compared to farmer practice.
• There is high probability to meet required soil moisture conditions for DSR sowing during early June.
• Supplemental irrigation for DSR crop establishment and to meet crop demands during dry periods can result in 5.8 t ha^−1^ in May and June sowings.

## 1 Introduction

Rice is the caloric mainstay for most of South Asia with the dominant share of total regional production emanating from the Indo Gangetic Plain (IGP) where it is commonly grown as a monsoon-season *kharif* crop in rotation with wheat on approximately 13.5 M ha (Timsina and Connor, 2001). The IGP is composed of the Western (WIGP - i.e. Punjab, Haryana, Western Uttar Pradesh) and Eastern zones (EIGP – i.e. Eastern Uttar Pradesh, Bihar, and West Bengal in India, the Terai of Nepal, and NW Bangladesh). Cropping systems in the WIGP tend to be highly mechanized and fully irrigated (Ladha et al., 2003a, 2003b, Gupta et al., 2003), with the resulting rice yields averaging around 4.0 t ha^−1^, providing approximately 75% of production in India and serving as a regional ‘grain basket’ (Dhillon et al., 2010). Nevertheless, stagnating yields (Ladha et al., 2003a,b, 2007) and intensive groundwater depletion (Humphreys et al., 2010) in the WIGP will pose serious challenges for achieving and sustaining the higher levels of production that will be required in coming decades as the South Asian population grows (Chauhan et al., 2013; Ladha et al., 2009). In contrast, staple crop management practices in the EIGP remain largely traditional and the region has tremendous potential for intensification since current rice yields average around 1.2 t ha^−1^ (Singh et al., 2009) despite abundant monsoon precipitation that provides an opportunity to sustainably extract more groundwater for irrigation with lower energy costs for pumping (Sikka et al., 2009).

Rice in the EIGP is commonly cultivated as a transplanted crop using long-duration varieties under rainfed conditions. Farmers typically delay nursery establishment until the onset of the monsoon, with transplanting occurring once there has been sufficient rainfall to enable soil puddling (Gopal and Kumar, 2010). Late onset of the monsoon combined with labor scarcity often delays rice transplanting and necessitates the use of older seedling, resulting in yield loss in rice and in also for the subsequent winter (*rabi*) crop due to delayed sowing (Cornish et al., 2015; Timsina and Connor, 2001). Dependence on monsoon onset for rice establishment also increases drought risk at anthesis and during grain filling if establishment is late (Cornish et al., 2015).

It has long been recognized that alternative rice establishment practices that facilitate timely establishment are necessary for significantly increasing the productivity of rice-wheat systems in the EIGP (Yamano et al., 2013). One such practice is DSR (Kumar and Ladha, 2011). In DSR, rice seeds are sown directly in the main field, avoiding the processes of nursery raising and transplanting. It also avoids soil puddling, requires less labor, and generally has a much lower irrigation requirement for crop establishment than transplanted rice (Kumar and Ladha, 2011; Laing et al., 2018). Timely rice establishment can also be achieved with DSR by taking advantage of pre-monsoon showers or applying a single light irrigation to create favorable soil moisture conditions for sowing and crop emergence. In addition to earlier establishment, a DSR crop matures 8–10 days faster by avoiding transplanting shock (Gopal and Kumar, 2010).

Despite considerable research and extension efforts since the mid-1990s, adoption rates for DSR in the EIGP remain extremely low. While weed management is a major constraint, the other primary impediment to broader-scale adoption is risk associated with crop establishment. Several studies report that the best time for sowing DSR is 10–15 days prior to the onset of the monsoon (Gopal et al., 2010; Kumar and Ladha, 2011). Delayed sowing past this window can jeopardize crop establishment by preventing field access for machinery due to wet soils or, alternatively, increase the risk of post-sowing seed or seedling mortality due to field inundation. If the timing of monsoon onset is accurately predicted, these risks can be avoided by planting 10–15 days beforehand. Unfortunately, the skill of monsoon weather forecasting is imperfect, and farmers must make decisions with considerable uncertainty about the timing and intensity of monsoon rainfall. So there is need to identify the probabilities of finding suitable conditions for DSR sowing over the possible range of monsoon scenarios using long term climate data. Early establishment of DSR can reduce the risk of crop failure from inundation, but it may also reduce crop yield potential (e.g. from lower solar radiation during grain filling) while increasing early-season irrigation requirements.

In general, in the EIGP, delayed or weak monsoon rains, late planting, and use of older seedlings often result in significant yield reductions. Even if transplanting is completed in a timely manner, irrigation may be required to avoid crop failure if monsoons onset is delayed or rains are unevenly distributed. By assessing resource requirements, median productivity, productivity stability across years, and tradeoffs between management objectives, this *ex ante* simulation study from central Bihar, India endeavors to identify agronomic interventions for both transplanted and DSR systems that may provide risk-reducing intensification pathways that can be selectively matched to farmer preferences in the context of existing production practices in the target region. This study will provide the probalistic understanding of sowing time, yield and yield stability under different improved agronomic interventions using long term climate data. Interventions assessed include time of planting, cultivar duration, supplemental irrigation, and seedling age (for transplanted systems).

## 2 Materials and methods

**Fig. 1:**
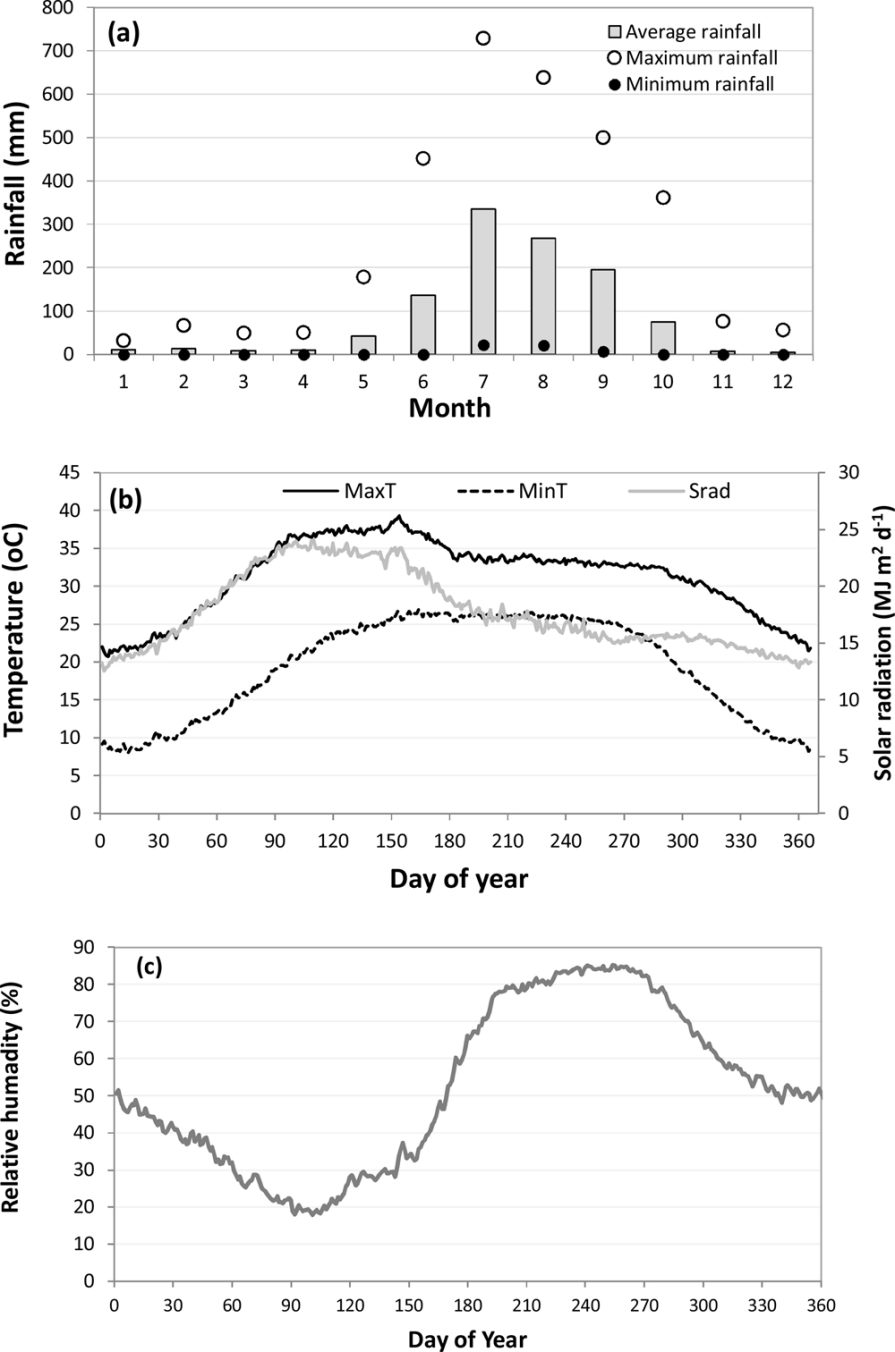
Long term (1970–2013) monthly mean, maximum and minimum rainfall (a), daily maximum and minimum temperatures and solar radiation, (b) and long term (1983–2013) average percentage relative humidity at Patna, Bihar (25.59 °N, 85.14 °E).

The study region is Patna District in Bihar State, eastern India. The climate in Patna (25.59 °N, 85.14 °E) is subtropical humid, with long-term (1970–2013) average annual rainfall of 1130 mm (range 630 to 1740 mm), 85–90% of which is received from June to September (Fig. 1a). Minimum daily mean temperatures are observed in December and January (< 10° C), with daily maximum temperature of around 40° C observed during the dry months of May and June (Fig. 1b). Solar radiation is high during dry months (April, May and June) but lower during monsoon months (July to September) (Fig. 1b). Relative humidity also varies during the year, with April has very low relative humidity (between 20%–30%) (Fig. 1c) and peaks during the monsoon months (around 80%). Three cropping seasons are present in Bihar: winter (*rabi*; November– March), summer (April–May), and rainy (*kharif*; June–November). Soils in the region are silt, silty loam, and sandy loam textures derived from alluvial deposition (Subash et al., 2011).

### 2.1 APSIM model (v7.7)

APSIM is a flexible modelling framework with linked sub-models representing crop, soil, and atmospheric processes that together simulate agricultural system performance (Holzworth et al., 2014). Example of crop sub-models include ORYZA for simulating rice growth and development. The crop sub-models simulate crop development, growth, water and nitrogen uptake, and are responsive to abiotic stress (e.g. water deficit, nitrogen deficit, aeration deficit). The rice crop sub-model is adapted from the ORYZA2000 model (Bouman and van Laar, 2006; Gaydon et al., 2012a, b). For the current study (and its antecedents), the rice sub-model in APSIM was modified to capture the effect of low temperature on rice spikelet sterility; details can be found in Balwinder-Singh et al., 2015. In the APSIM model, abiotic stresses are calculated and represented by different indices. For example, *lstrs* is the drought stress factor (dimensionless) for leaf rolling, with values ranging from one to zero (value = 1 no stress, 0= maximum stress). In rice crop leaf rolling is first visible sign when plant is subjected to drought stress and we used this as indictor (lstrs less than 0.8) to apply irrigation to avoid any growth penalty in irrigation scenarios. We calculated average value of drought stress index (lstrs) during the vegetative period (transplanting to anthesis) and during the reproductive period (anthesis to maturity) to compare the severity of drought during these stages under various simulation experiments.

### 2.2 Model parameterization, calibration and validation

The APSIM model was parameterized, calibrated and its performance verified for two rice varieties: a long-duration rice cultivar (MTU7029, 150 day duration); and a medium-duration rice hybrid (Arize6129, 125 day duration), grown on a silty loam soil as part of production-scale field experiments coordinated by CSISA (Cereal Systems Initiative for South Asia – www.csisa.org) in 2014 and 2015 at the Indian Council for Agricultural Research- Research Complex for Eastern Region (ICAR-RCER) in Patna, Bihar. Each variety was established at five different transplanting dates (in 2014: 1 July, 15 July, 8 August, 19 August, 1 September ; in 2015: 1 July, 15 July, 30 July, 14 August, 30 August). In all the treatments, 30-day old seedlings were transplanted at 33 hills m^−2^ with 2 seedlings per hill. All the crops were provided with recommended irrigation and nutrients (e.g. 150 kg N ha^-1^). Field data from the 15 July transplanting date in 2015 was used for APSIM calibration and parameterization. We selected this date because it falls in rice transplanting window when maximum yield was observed. Genetic coefficients were estimated by iteratively varying the values of the coefficients to produce a close match between simulated and measured phenology, grain yield and total biomass. The genetic coefficients of both the rice varieties are presented in Table 1. Using the same genetic coefficients for the MTU7029 rice cultivar, APSIM was also evaluated for DSR with data from an adjacent field experiment from the 2014 growing season where the crop was sown during first week of June and supplied with recommended nitrogen and irrigation water.

**Table 1:** Genetic coefficients of rice varieties used in simulations.

Crop development stage	Genetic coefficients	
	MTU7029 (Long duration)	Arize6129 (Short duration)
Development rate in juvenile phase (oCd^−1^)	0.000593	0.000700
Development rate in photoperiod-sensitive phase (oCd^−1^)	0.000596	0.000400
Development rate in panicle development (oCd^−1^)	0.000550	0.000700
Development rate in reproductive phase (oCd^−1^)	0.002050	0.002050
Photoperiod sensitivity (h-1)	0.2	0.3
Maximum optimum photoperiod (h)	11.2	11.4
Spikelet growth factor (no kg-1)	64000	64900
Fraction shoot dry matter partitioned to the panicles as a function of 1.0, 1.2 and 2.50 development stage	0.3, 0.6, 0.6	0.5, 1.0, 1.0

Simulated model output was compared with observed values from all treatments in the two experiments. Model performance was assessed using the absolute and normalized root mean square error (RMSE), and the coefficient of determination (r^2^) of the regression of observed against simulated values forced through the origin. Smaller RMSE (of the same, or less, order of magnitude as experimental standard deviations) and high r^2^ values indicate good agreement between model outputs and observed values.

(1) $$RMSE=\sqrt{\frac{\sum_{i=1,n}{\left(S_{i}-O_{i}\right)}^{2}}{n}}$$

Where S_i_ and O_i_ are predicted and observed values, respectively, and n is the number of observations

(2) $$\text{N}\text{o}\text{r}\text{m}\text{a}\text{l}\text{i}\text{z}\text{e}\text{d}\;\text{R}\text{M}\text{S}\text{E}\;(\text{\%})=\left(\frac{\text{A}\text{b}\text{s}\text{o}\text{l}\text{u}\text{t}\text{e}\;\text{R}\text{M}\text{S}\text{E}}{\text{M}\text{e}\text{a}\text{n}\;\text{o}\text{f}\;\text{t}\text{h}\text{e}\;\text{o}\text{b}\text{s}\text{e}\text{r}\text{v}\text{e}\text{d}\;\text{v}\text{a}\text{l}\text{u}\text{e}\text{s}}\right)\times 100$$

The soil parameters are based on the properties of a field site at ICAR-RCER, Patna (Laik et al., 2014) and are presented in Table 2. The soil has a plant available water capacity (PAWC) of 92 mm at the 0–60 cm soil depth and a PAWC of 235 mm at 0-150 cm. The stage 1 soil evaporation parameter (U) was set to 12 mm, and the stage 2 parameter (*cona*) was set to 3 mm, based on the values used by Balwinder-Singh et al. (2011) for silty loam soil. Saturated percolation rate (ks) was set to 12 and 3 mm d^−1^ for non-puddled (i.e. for DSR systems) and puddled soil (for transplanted system), respectively, based on data from Humphreys et al. (2008). Weather data (maximum and minimum temperature, sunshine hours) from the 2014 and 2015 seasons were collected from the meteorological station which is one km away from the experiment site but rainfall was collected on site. Solar radiation was calculated from daily sunshine hours using the Angstrom Formula:

(3) Rs = Ra(a + b)(n/N))

where: Rs is total radiation received at the earth’s surface; Ra is the extra-terrestrial radiation at the edge of the atmosphere and calculated for each day from latitude and day of year; n is the sunshine hours; N is the day length (hours); a and b are the Angstrom coefficients and were calculated from latitude using equations suggested by Bandyopadhyay et al. (2008).

**Table 2:** Physical properties of the silty loam soil used in the simulations.

Soil layer depth (cm)	LL (cm^3^ cm^−3^)	DUL (cm^3^ cm^−3^)	SAT (cm^3^ cm^−3^)	BD (gcm^−3^)	pH
0-15	0.050	0.200	0.390	1.190	6.7
15-30	0.060	0.210	0.410	1.230	7.0
30-60	0.070	0.230	0.380	1.300	7.3
60-90	0.100	0.240	0.380	1.350	7.6
90-120	0.080	0.250	0.360	1.300	7.7
120-150	0.120	0.280	0.360	1.320	7.8

LL—volumetric water content at lower limit. DUL—drained upper limit. SAT—saturation. BD—bulk density.

### 2.3 *Ex ante* simulation experiments

The calibrated APSIM model was used to conduct a multi-criteria assessment of different agronomic interventions in transplanted and DSR rice systems under long term simulations using 44 years (1970–2013) of historical weather data from the meteorological station at ICAR-RCER in Patna, Bihar. System performance was judged in terms of grain yield, yield stability, irrigation requirements, and water productivity. Water productivity was computed with respect to irrigation (WP_I_) in terms of kg grain ha^−1^ mm^−1^:

(4) WP_I_ (kg ha^−1^ mm^−1^) = Grain yield (kg ha^−1^)/Total irrigation amount (mm)

#### 2.3.1 Experiment #1: simulating the effect of transplanting date under irrigated conditions

APSIM was used to compare climatically-determined potential yield of MTU7029 and Arize6129 rice cultivars for 15 nursery sowing dates from 1 May to 14 August at 7-day intervals. In turn, 15 transplanting dates were simulated using 30 day-old seedlings, for example, a nursery established on 1 May was transplanted on 31 May. The simulations were run under non-limiting nitrogen and water conditions. The soil was flooded one day before transplanting on the day of puddling; the amount of water applied was sufficient to fill the top two soil layers (0–30 cm) to saturation plus an additional 50 mm of water to create a surface pond for puddling. Recommended irrigation management for transplanted rice involves keeping the field flooded for the first two weeks after transplanting, followed by alternate wetting and drying (AWD, i.e. irrigating once the topsoil becomes dry). Therefore, a pond depth of 50 mm was maintained for the first 15 days after transplanting by irrigating daily when needed. After that, the crop was irrigated two days after the disappearance of ponded water; the amount of water added at each irrigation was that required to fill the top two soil layers (0–30 cm) to saturation plus an additional 50 mm water to create surface ponding. Irrigation of rice ceased one week before crop maturity, and the rice was harvested eight days after physiological maturity to allow time for grain moisture content to decline to less than 20%.

#### 2.3.2 Experiment #2: Agronomic interventions for typical farmer-managed transplanted rice cultivation (fTR)

In this experiment, we evaluated the performance of a transplanted crop with management rules designed to mimic those commonly used by farmers in the EIGP (Cornish et al., 2015; Umashaanker, 2019). In the simulation set-up, the nursery sowing window extended from 15 May until 15 August, with the nursery established only when cumulative rainfall received was equal to or greater than 50 mm over three consecutive days during the sowing window. There was no rice nursery established or crop sown if these conditions were not met by 15 August. The cultivar MTU7029 was used to represent farmer preferences for long-duration varieties (i.e. 150 day duration). The rice crop was transplanted when seedlings were at least 21 days old and when surface water ponding exceeded 50 mm as a result of heavier rainfall, which represents minimum requirements for soil puddling and subsequent transplanting (Cornish et al., 2015). The days between nursery sowing and transplanting constitute seedling age. The crop was grown under rainfed conditions and supplied with 150 kg N ha^−1^ fertilization, 50% N at time of transplanting and 50% after 3 weeks of transplanting

Additional simulations were then run to identify ways to improve the performance of prevailing farmer-managed systems (fTR). From the base fTR simulations, three experiments were conducted to assess the impact of changing single management factors within fTR:

1) *Cultivar*: in place of the long-duration MTU7029, a medium-duration hybrid (Arize6129) was cultivated.
2) *Supplement irrigation*: in place of rainfed management, irrigation water was applied whenever the drought stress index ‘*lrstrs*’ fell below 0.8 post-transplanting; 0.8 was chosen because growth penalties in APSIM start at that value. At each irrigation, sufficient water was added to saturate the top 30 cm of soil plus an additional 50 mm water to create surface ponding.
3) *Seedling age*: in place of variable seedling ages, 30-day (‘appropriately aged’) seedlings were transplanted as soon as sufficient rain had been received at the onset of the monsoon and surface-water ponding exceeded 50 mm, a treatment we term ‘appropriate aged seedling’ transplanted rice cultivation. In fTR, nurseries were sown with monsoon onset, but in appropriate seedling age scenario transplanting occur with onset of monsoon.

#### 2.3.3 Experiment #3: Optimum planting dates for rainfed DSR

In this experiment, rainfed DSR performance was assessed within the 1 May to 31 August sowing window. DSR establishment was initiated when soil moisture in the 0–15 cm soil layer was between 40–80% of field capacity for three consecutive days. The DSR crop using long duration rice variety (MTU7029) was sown at a density of 150 plants m^−2^ with row spacing of 20 cm. Plant density was reduced if heavy rains occurred after sowing based on field observations of seedling mortality rates by the authors. We estimate that ponding for two, three or four days during the first five days after sowing reduced plant density by 15, 40 and 75%, respectively. If ponding persisted for five days, the crop failed and was not re-sown. In another scenario, the long duration cultivar (MTU7029) was replaced with medium duration hybrid (Arize6129) while keeping other management practices the same. No DSR crop was sown if field remains wet (soil moisture more than 80% of field capacity) during this window to reflect the condition of non-accessibility of field to sowing machinery.

#### 2.3.4 Experiment #4: establishing the benefits of irrigating DSR

In the irrigated DSR scenario, 11 sowing dates from 1 May to 31 August were evaluated at seven days intervals. The simulation set-up was same as in Experiment #3, with sowing done only when soil moisture in the 0–15 cm soil layer was between 40–80% of field capacity for three consecutive days. If soil moisture was less than 40% on first day of the sowing window, pre-sowing irrigation was applied and sowing was done when soil moisture rose to 40–80% of field capacity. No DSR crop was sown if soil moisture was more than 80% of field capacity during this window. Post-sowing supplementary irrigation was applied whenever the drought stress index (*lrstrs*) was less than 0.8. At each irrigation, the amount of water added was the depth required to fill the top 30 cm of soil to saturation plus an additional 50 mm water to create surface ponding.

#### 2.3.5 Statistical analysis

Results from the experiments were evaluated through analysis of variance (ANOVA) using Genstat (v 13.0) keeping sowing date, variety, supplemental irrigation, and seedling age as factors contrasted to baseline farmer practice (fTR) as appropriate with years serving as replicates. The differences between treatments were evaluated for significance using least significant difference (LSD) at the 95% confidence level.

## 3 Results and discussion

### 3.1 Model evaluation

**Fig. 2:**
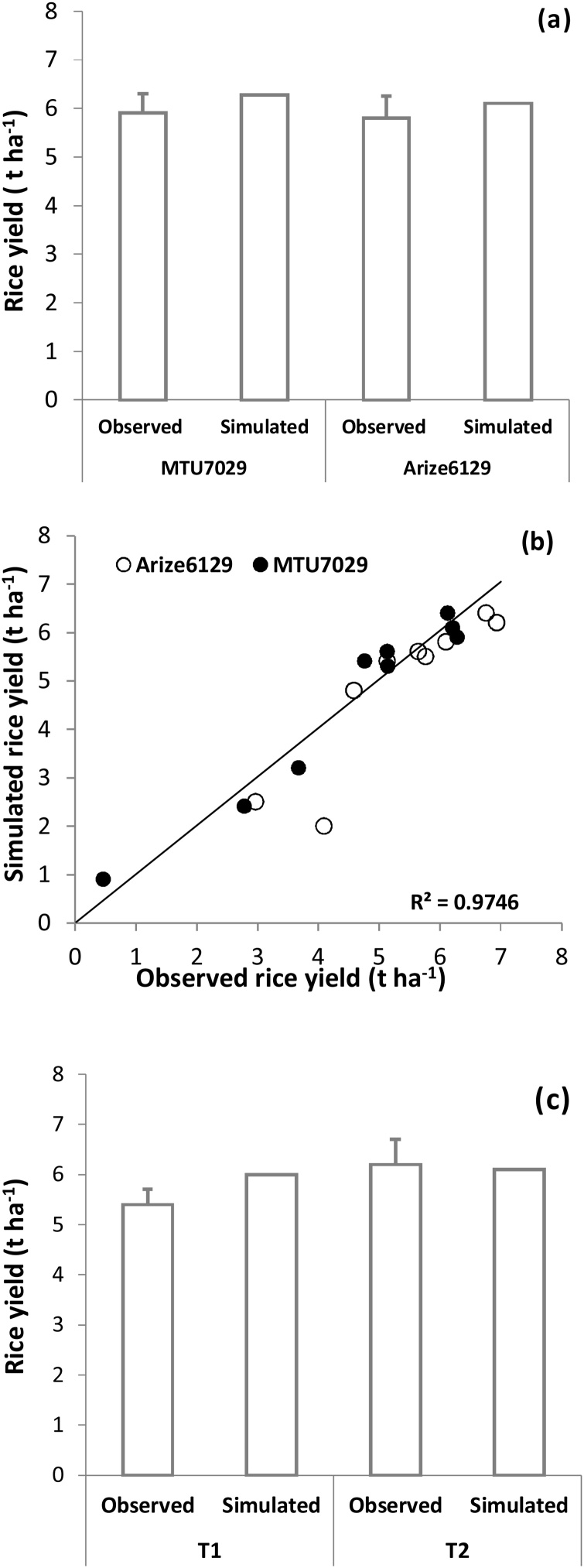
(a) Simulated and observed grain yield in calibration dataset, (b) Simulated and observed grain yield in validation dataset, (c) Simulated and observed rice yield during 2014 rice season in DSR treatments (validation). T1- Rice-wheat system, T2-Rice-maize system.

The calibrated model performed well, as evidenced by its ability to predict yields of transplanted rice varieties of different maturity class under different sowing dates with an r^2^ value of 0.97 under validation dataset (Fig. 2b). There was good agreement between simulated and observed yield for all treatments with a low absolute RMSE (0.03 t ha^−1^) and normalized RMSE (5%). The model was able to predict declining yield trends with delay in transplanting after changing the *cttmax* (cooling degree-day threshold temperature) from 22° C to 28° C (Balwinder-Singh et al., 2015). Similarly, model performance was good for simulating DSR rice yields (Fig. 2c), implying that the same crop genetic coefficients can be used to accurately simulate DSR and transplanted systems in APSIM. Crop phenology was also well-predicted, with good agreement between simulated and observed dates of both 50% flowering and physiological maturity for the different sowing date treatments in each year. The simulated 50% flowering occurred within two to three days of observed date, and simulated physiological maturity occurred within three to four days of observed maturity. Evidence of the good performance of APSIM-Oryza and the standalone ORYZA model under transplanting and DSR conditions in the IGP is also provided by Balwinder-Singh et al., 2015, and Sudhir-Yadav et al. (2001a, 2011b, 2012). Furthermore, Gaydon et al. (2017) evaluated APSIM-Oryza for 361 rice datasets (265 transplanted and 96 DSR) across South and Southeast Asia under a range of agronomic, water management, and production practices and found APSIM performed well with r^2^ = 0.83 and RMSE = 1.14 t ha^−1^. These results demonstrate the robustness of model performance in the region and its reliability for use in *ex ante* assessements.

### 3.2 Experiment #1: simulating the effect of transplanting date under irrigated conditions

**Fig. 3:**
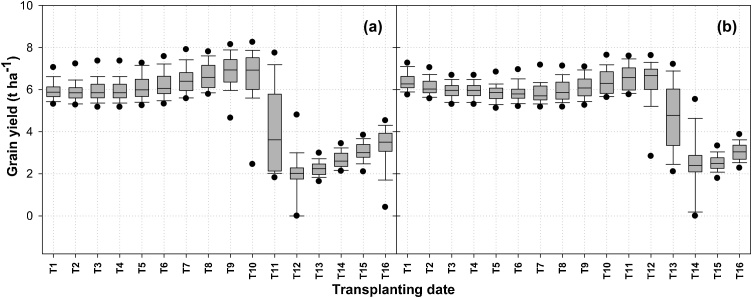
Effect of transplanting date on simulated potential grain yield of (a) MTU7029, (b) Arize6129 rice variety on a silty loam soil under over 43 years (1970–2013). T1 = 31May, T2 = 7June, T3 = 14June, T4 = 21June, T5 = 28June, T6 = 5 July, T7 = 12 July, T8 = 19 July, T9 = 26 July, T10 = 2 Aug, T11 = 9 Aug, T12 = 16 Aug, T13 = 23 Aug, T14 = 30 Aug, T15 = 7 Sept, T16 = 14 Sept. Vertical shaded bars are 25th-75th percentiles; whisker caps are 10th and 90th percentiles and black dots 5th and 95th percentiles.

Small yield increases were observed as transplanting dates shifted from 31 May towards 2 August for a long-duration cultivar, and from 31 May to 16 August for the medium-duration hybrid (Fig. 3a and b) but the trend was not significant. The highest median yield for a long-duration cultivar was 6.8 t ha^−1^ when transplanting occurred between 26 July and 2 August (Fig. 3a). Early August transplanting of the long-duration cultivar resulted in low yields in many years, and further delay to mid-August resulted in highly significant yield declines to a median of 2.2 t ha^−1^. For the medium-duration rice hybrid, the highest median yield of 6.6 t ha^−1^ was observed when seedlings were transplanted on 9 August or 16 August (Fig. 3b), with yields declining sharply thereafter. Rice yields for both long-duration variety and medium-duration hybrid were statistically similar at all transplanting dates except 9 August, 16 August, and 23 August where clear advantages were apparent for the hybrid, suggesting a broader window of opportunity for high yields and resilience to planting date variations than the current farmer practice of planting long-duration cultivars. The best combination of high and stable yields was observed at the 19 July and 2 August transplanting dates for a long and medium-duration cultivars, respectively.

The results from the simulations are consistent with the findings from the field and the modeling studies in India which suggest that the yield of transplanted rice is relatively stable over a wide range of transplanting dates in June and July, but decline with late planting (e.g. Hira and Khera, 2000; Jalota et al., 2009a; Khepar et al., 1999; Basu et al., 2014; Chopra et al., 2003; Singh et al., 2012). This study adds to that body of knowledge by demonstrating that the influence of sowing date on yield and yield stability varies significantly with cultivar duration. For the long-duration cultivar, delaying transplanting to early August resulted in considerable yield decline in some years, and a further delay to mid-August resulted in low yields in all years (Fig. 3a). However, the medium-duration cultivar maintained high yield levels up to mid-August transplanting. At the process level, low yields resulting from later transplanting were associated with exposure to cooler temperature (< 28 °C average daily temperature) during the reproductive and grain filling stages which resulted in high spikelet sterility and even crop mortality in very cool years, consistent with the results of the field experiments of Nagarajan et al. (2010) and simulation results of Balwinder-Singh et al. (2015). Conversely, very early transplanting of long-duration cultivars had comparatively low yield levels as compared to the optimal mid-July transplanting because of lower levels of solar radiation during reproductive growth. This dynamic was not evident for the medium-duration cultivar which had comparatively high yields with early transplanting. These results demonstrate the risk-reduction and performance stability that can be achieved through adoption of medium-duration hybrids that are well-adapted to both early and later transplanting.

### 3.3 Experiment #2: Agronomic interventions for typical farmer-managed transplanted rice cultivation (fTR)

**Fig. 4:**
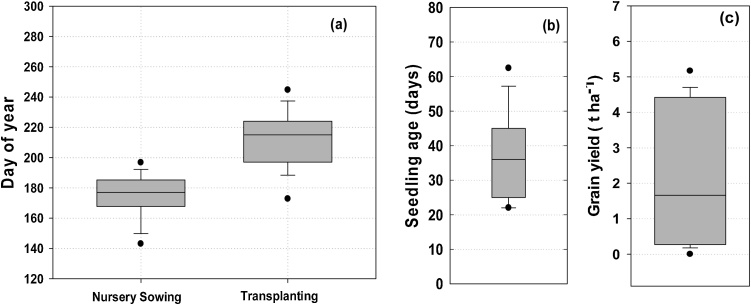
(a) Nursery sowing day and transplanting day under rainfed farmers; transplanted rice management, (b) seedling age in days, and (c) rice grain yield (t ha^−1^) over 44 years (1970–2013). Vertical shaded bars are 25th-75th percentiles; whisker caps are 10th and 90th percentiles and black dots 5th and 95th percentiles.

In EIGP, traditional rice management practices result in late rice establishment, and use of older seedling due to asynchrony between nursery sowing and the time at which the main field is ready for transplanting. Using the farmer-based decision rules described in section #2.3.2, APSIM simulations showed that rainfall-dependent nursery sowing dates have large variability: the median nursery sowing date was 26 June (177 day of year (DOY)) but ranged from 23 May (143 DOY) to 29 July (210 DOY) (Fig. 4a). Similarly, seedling age at transplanting time was also variable, ranging from 22 to 79 days, with a median of 38 days (Fig. 4b). In only 42% of the years was seedling age within the recommended 30-day maximum typically required to maintain yield potential.

Transplanting dates were also variable, ranging from 24 June (175 DOY) to 20 September (263 DOY), with a median of 2 August (214 DOY) (Fig. 4a). The variation in transplanting dates was higher than nursery sowing dates, with a standard deviation of 24 days for transplanting as compared to 17 days for nursery sowing. There were five years out of 43 when crop establishment did not occur (i.e. field remained fallow) because simulated soil moisture conditions were never conducive for transplanting within the planting window.

**Fig. 5:**
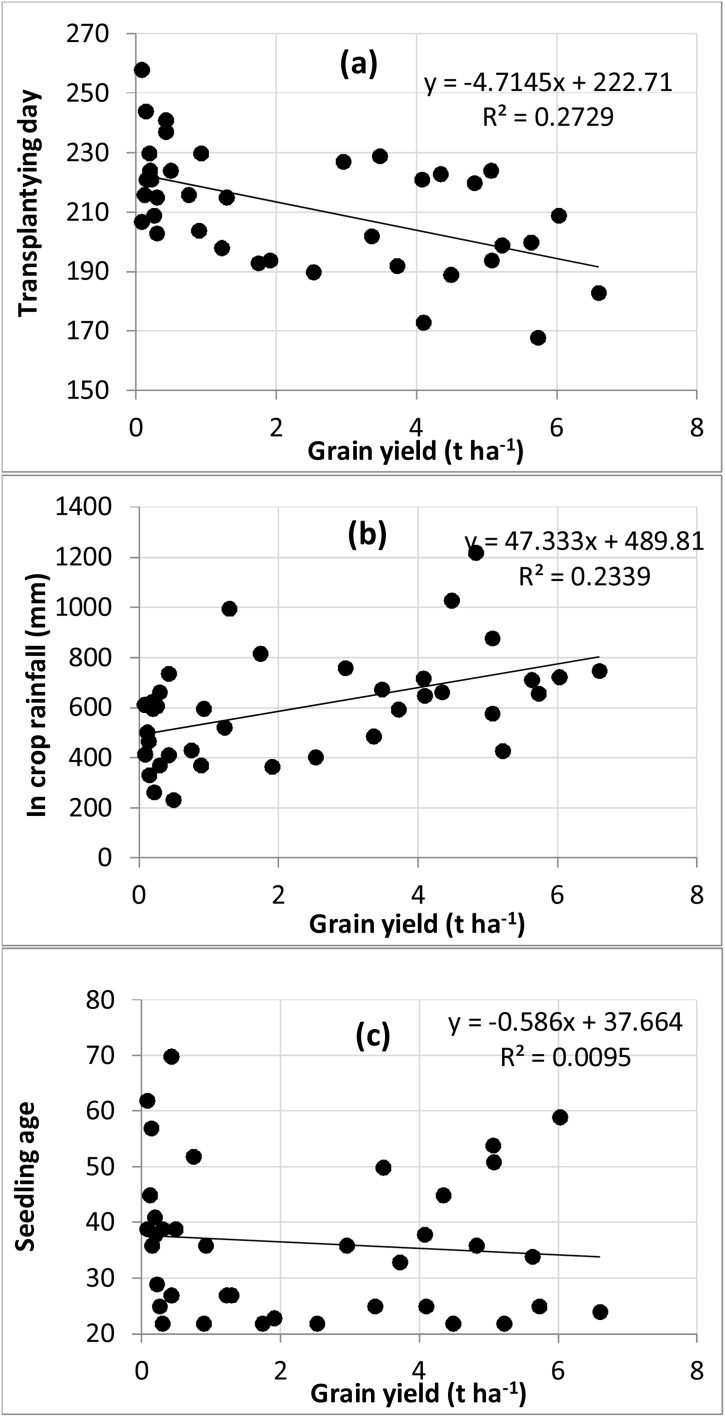
Relationship between rice grain yield and (a) transplanting day (Julian day of year), (b) monsoon rainfall, (c) seedling age under farmers’ management practice (fTR).

The uncertainties in rice crop management under prevailing farmer practice (i.e. rainfed cultivation in transplanted systems with long-duration varieties) have significant implications for grain yields over the long term. The median yield was 1.6 t ha^−1^, ranging from 0 to 5.8 t ha^−1^ (Fig. 4c) with a standard deviation of 2.1 t ha^-1^ (Table 3). In only 58% of years did yields exceed 1 t ha^−1^. High yields were simulated in years where timely transplanting with optimum aged seedlings coincided with ample monsoon rains. For example, the highest yields simulated with farmer management practices were in 1995 when the crop was transplanted on 2 July (183 DOY) with 24-day seedlings and the total monsoon rainfall was 1130 mm. The lowest yields were associated with delayed transplanting, old seedlings, and post-establishment drought. Approximately 50% of the yield variability was explained by transplanting date and monsoon rainfall (Fig. 5a, b & c).

**Table 3:** Median yield, number of irrigation events, drought stress index and standard deviation of grain yield under farmer practice and under various risk-reduction strategies over 44 years (1970–2013).

Rice establishment method + risk reducing strategy	Median yield (t ha^−1^)	Standard deviation in grain yield (t ha^−1^)	Median number of irrigation events	Median drought stress index during grain filling period
fTR	1.6	2.1	–	0.41 (39)**
fTR + supplemental irrigation	3.2	1.4	4 (1-10)*	0.86 (0)
fTR + short duration rice	3.2	2.2	–	0.64 (18)
fTR + supplemental irrigation + short duration rice	4.6	1.0	2.5 (0-8)	0.88 (0)
fTR + appropriate aged seedling	3.4	1.9	–	0.65 (22)

fTR-Farmer practice of puddled transplanted rice.

iTR- Improved puddled transplanted rice with 30 days old seedling.

* Figures in parentheses represent the range of irrigation numbers over 44 years.

** Figures in parentheses represent the number of years out of 44 years when average drought stress index during grain filling period was less than 0.80.

Applying supplemental irrigation significantly (p < 0.05) increased median yields by 1.6 t ha^−1^ to 3.2 t ha^−1^ with the SD decreasing from 2.1 t ha^−1^ to 1.4 t ha^-1^ in contrast to fTR. The median number of irrigations required was four but ranged from one to ten depending upon the amount and temporal distribution of monsoon rainfall. Replacing the commonly planted long-duration cultivar in fTR with a medium-duration hybrid resulted in similar median yield gain to that achieved with supplemental irrigation but no reduction in yield variability. When both supplemental irrigation and a medium-duration hybrid were combined, average yield increased to 4.6 t ha^−1^ with further reductions in yield variability beyond that achieved with supplemental irrigation (SD = 1.0 t ha^−1^) (Table 3), suggesting a strong synergy between these practices.

**Fig. 6:**
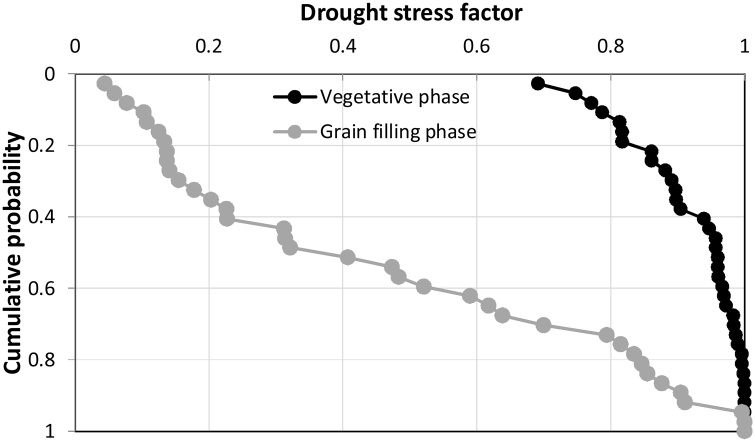
Drought stress factor (lstrs) during vegetative and reproductive phase over 44 years (1970–2013) under farmer practice (fTR) using the long duration variety (MTU7029). (drought stress index value = 1 no stress, 0= maximum stress).

Late transplanting in fTR pushes the crop growth beyond the main monsoon period, and in 60% of years the simulated drought stress index lrstrs was < 0.2 (severe) during grain filling (Fig. 6). The yield advantage achieved by replacing the long-duration cultivar with a medium-duration hybrid was mainly because of a reduced risk of drought stress at grain filling (index value was 0.64 under the medium duration hybrid and 0.41 under the long duration rice variety); combining supplemental irrigation with the medium-duration hybrid increased the index to 0.86.

We then assessed the benefits of transplanting appropriately-aged seedling. By transplanting 30-day old seedlings at the onset of the monsoon (seedling age scenario), the median productivity for a long-duration cultivar increased by 1.8 t ha^−1^ compared to fTR (Table 3) with the median transplanting date advancing to 13 July 13 as compared to 2 August under fTR. Relatively early transplanting in appropriate seedling age scenario partly reduced drought risk, but yield variability was little changed (Table 3).

Our results are consistent with a survey study conducted by Singh et al., 2001, Singh et al., 2002 in Patna District in which the authors found that only 30% of farmers transplanted rice before end of July during the 1998 rice season. Asynchrony between the timing of nursery establishment and transplanting in the main field is reason for using of aged seeding at time of transplanting and this is a persist contributor to yield gaps. Our results showed that transplanting appropriate seedlings can result in 1.8 t ha^−1^ higher yield than fTR. Timely availability of appropriate aged seedlings is possible through community nursery approach. The technique involves raising a staggered community nursery under assured irrigation in the village at an interval of two weeks and seedlings are available in market for farmers at the time of transplanting. In Bihar, State Department of Agriculture has launched a scheme for promoting farmer managed community nurseries under assured irrigation to make available paddy seedlings for transplanting to meet contingent situations.

### 3.4 Experiment #3: Optimum planting dates for rainfed DSR

**Fig. 7:**
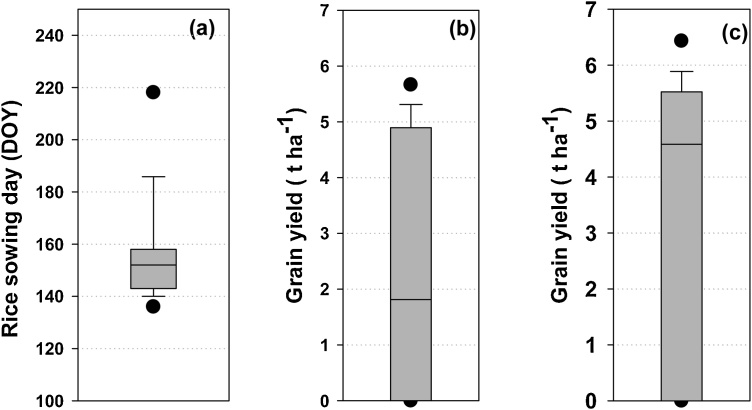
(a) Sowing date for the long duration and short duration rice variety, (b) rice grain yield under a long duration variety (MTU7029), (c) under a short duration rice variety (Arize6129) for rainfed DSR over 44 years (1970–2013). Vertical shaded bars are 25th-75th percentiles; whisker caps are 10th and 90th percentiles and black dots 5th and 95th percentiles.

Under rainfed conditions, simulations suggest considerable variability in the date at which DSR can first be sown, ranging from 19 May (139 DOY) to 10 August (222 DOY) with median sowing date of 2 June (153 DOY) (Fig. 7a). In three years, sowing was not possible as soil moisture criteria were not met. The median grain yield using the long-duration rice variety was around 2 t ha^−1^ (Fig. 7b) and ranged from 0 to 6.0 t ha^−1^. In 40% of the years, the crop experienced an extended dry spell in the early vegetative phase which resulted in crop failure or low yields (< 1.0 t ha^−1^). In years with sowing after mid-June (20% of all years), the risk of terminal drought stress increased during the grain filling period which also resulted in low yields. The resilience strategy of cultivating a medium-duration hybrid significantly lowered drought risk, and median grain yield more than doubled to 4.5 t ha^−1^ with this single intervention in DSR systems (Fig. 7c).

Gopal et al. (2010) and Kumar and Ladha (2011) also suggested that the best time for sowing rainfed DSR in India is about 10–15 days prior to the full onset of the monsoon which is close to our simulated results (median sowing date of 2 June). Based on long term rainfall data (1960–2017) at Patna, average monsoon onset date is 18 June (Subash et al., 2011). Based on our results, sowing earlier than this creates high risks of crop failure, while delayed sowing increases the risk of encountering wet soil conditions that prevent machinery access for crop establishment or, alternatively, may result in poor stand establishment due to seed or seedling mortality.

### 3.5 Experiment #4: establishing the value of irrigating DSR

**Fig. 8:**
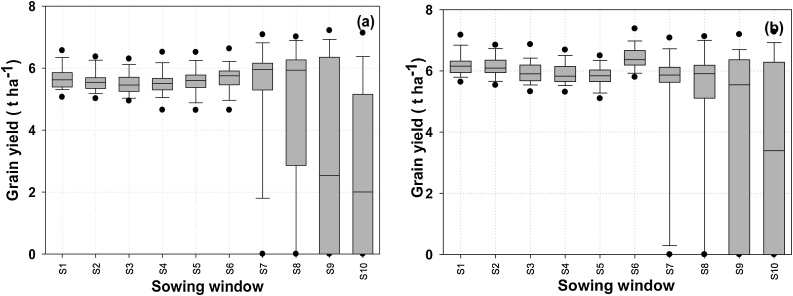
The effect of supplement irrigation on variability of DSR grain yield (t ha^−1^), (a) long duration variety (MTU7029), (b) short duration variety (Arize6129) under different sowing windows (S1-S10). Sowing window start dates are 1 May (S1), 8 May (S2), 15 May (S3), 22 May (S4), 29 May (S5), 5 June (S6), 12June (S7), 19 June (S8), 26 June (S9), 3 July (S10). Vertical shaded bars are 25th-75th percentiles; whisker caps are 10th and 90th percentiles and black dots 5th and 95th percentiles.

Supplement irrigation can minimize the drought risk and also ensure timely planting of DSR. Simulation results suggest that yields with supplemental irrigation (Fig. 8a and b) are significantly higher and much more stable compared to those under rainfed DSR (Fig. 7b and c) with both a long and a medium-duration rice cultivar. With the long-duration cultivar, yields were around 5.8 t ha^−1^ for May to mid-June sowing (S1 to S7) and SD was around 0.4 t ha^−1^. After mid-June, yield variability increased (SD 2.6 t ha^−1^) due to a higher probability of missing DSR sowing due to non-accessibility of the field for machinery and, for crops that are established, low temperature stress during grain filling. In early sowings, the probability of achieving relatively high yield was better for the medium-duration hybrid (Fig. 8b); a median yield of around 6 t ha^−1^ was maintained up to the S8 sowing window compared to S6 with the long-duration cultivar.

**Fig. 9:**
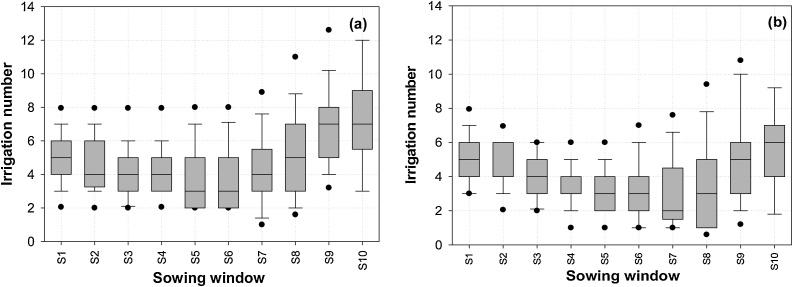
The number of irrigation events under supplemental irrigation scenario, (a) long duration variety (MTU7029), (b) short duration variety (Arize6129) under S1-S10 sowing windows. Sowing window start dates are 1 May (S1), 8 May (S2), 15 May (S3), 22 May (S4), 29 May (S5), 5 June (S6), 12June (S7), 19 June (S8), 26 June (S9), 3 July (S10). Vertical shaded bars are 25^th^-75^th^ percentiles; whisker caps are 10^th^ and 90^th^ percentiles and black dots 5^th^ and 95^th^ percentiles.

**Fig. 10:**
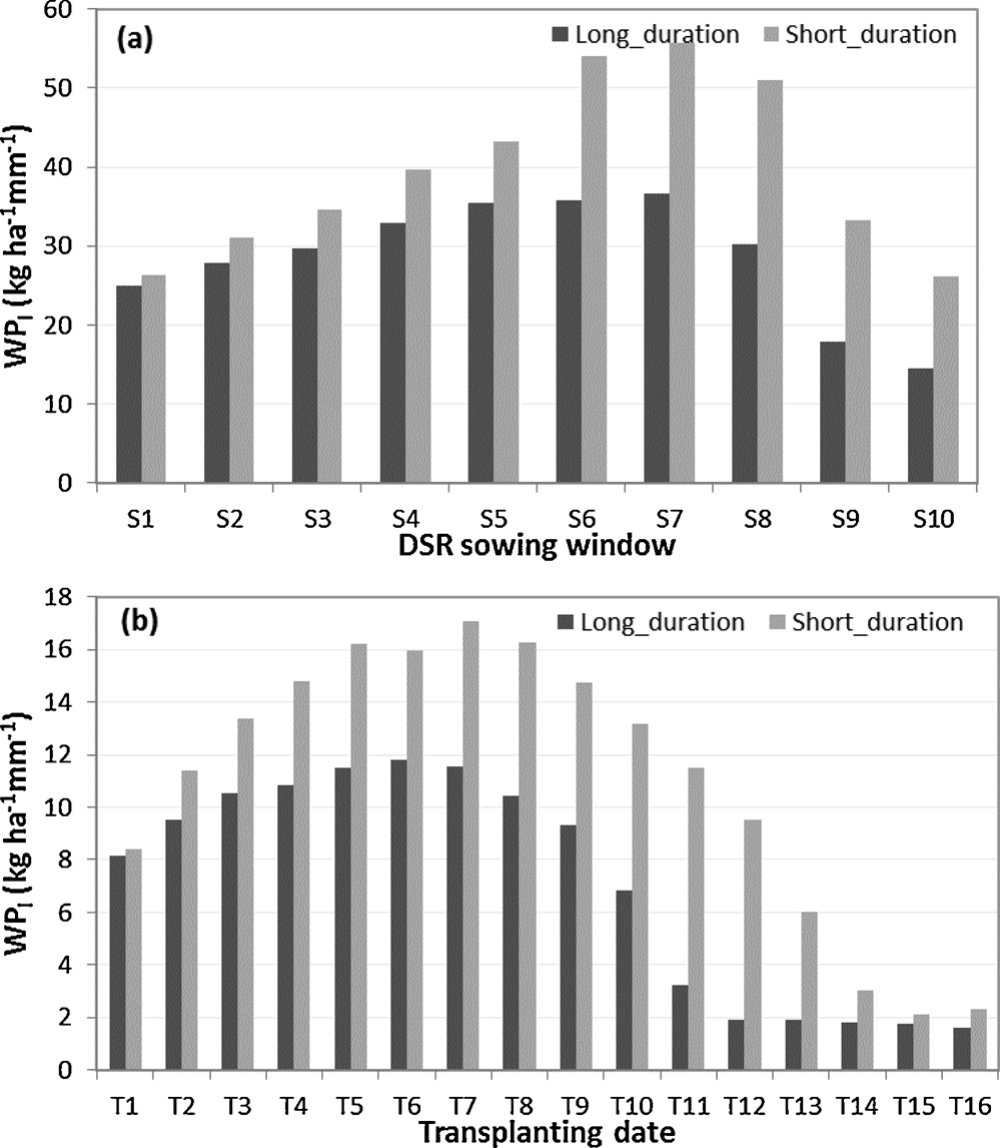
Average water productivity based on irrigation amount (WP_I_) (kg ha^−1^ mm^−1^), (a) under irrigated DSR for a long and a short duration rice variety under different sowing windows (S1-S10), (b) under irrigated PTR for a long and a short duration rice variety under different transplanting dates (S1-S16). DSR sowing window start dates are 1 May (S1), 8 May (S2), 15 May (S3), 22 May (S4), 29 May (S5), 5 June (S6), 12June (S7), 19 June (S8), 26 June (S9), 3 July (S10). Transplanting dates are as T1 = 31May, T2 = 7June, T3 = 14June, T4 = 21June, T5 = 28June, T6 = 5 July, T7 = 12 July, T8 = 19 July, T9 = 26 July, T10 = 2 Aug, T11 = 9 Aug, T12 = 16 Aug, T13 = 23 Aug, T14 = 30 Aug, T15 = 7 Sept, T16 = 14 Sept.

Although grain yield was similar for all sowing dates until mid-June under long-duration varieties, the number of irrigation events decreased from early May to mid-June, then increased again thereafter (Fig. 9a). Similar trends for the medium-duration hybrid were observed, but with fewer irrigations generally required (Fig. 9b). For both cultivars, WP_I_ was highest with mid-June sowing (Fig. 10a). Similar trends were observed for irrigated transplanted conditions for both long duration and short duration rice varieties (Fig. 10b), however WP_I_ was low under transplanted crop as compare to DSR crop which confirm that DSR is more water efficient practice as compared to transplanting. Early-May sown crops were exposed to higher evaporative demand and lower early rainfall, hence required more frequent irrigation. The increase in irrigation for the long-duration cultivar was because crop maturity commonly extended beyond the cessation of monsoon rains.

Our estimates of irrigation requirements and water productivity are consistent with the findings of field and other simulation experiments in transplanted rice which show significantly lower irrigation input and higher WP_I_ with shorter-duration cultivars (Amarasingha et al., 2015; Brar et al., 2012; Mahajan et al., 2009; Jalota et al., 2009b). For irrigated DSR with a short-duration variety, the optimum sowing window is early to mid-June (S5-S6) when yield is higher and stable with lower irrigation requirements and higher water productivity. Earlier sowings may have high yield but require more irrigation water, hence have low WP_I_. Delaying planting after late June is generally not possible because of field access issues, high irrigation requirements, and high risk of cold injury. In Bihar where diesel pump sets are commonly used as the power source for irrigation and energy costs are high, there is a general imperative to increase WP_I_ and hence to sow DSR crops by early June. Farmers irrigate their rice crops (average 2–3 irrigations, Urfels, unpublished data) but irrigation intensity is low because irrigation is expensive. However, due to shallow groundwater levels and state government subsidy policies on installing pump sets, access to irrigation is widespread now and 61% of sown area in Bihar is irrigated (Kishore et al., 2014), however, only 46% of the available groundwater is being withdrawn for agriculture (Najmuddin et al., 2018). Almost all groundwater irrigators in Bihar irrigate from diesel pump sets—own or rented because there is no electricity in half of the villages of the state, and even in the other half, the power supply is extremely poor and unreliable. With poor electricity, diesel becomes an expensive option for the farmers in Bihar. Irrigation intensification will take place only if the variable cost of irrigation comes down and through improvement in electricity infrastructure and supply of cheap electricity for agriculture as happened in north Indian states. Recently government launched new schemes to electrifying each village by year 2022. Bihar state government plans to increase the present irrigation intensity from 83% to 158% by year 2017 and 209% by year 2022 by investing into to improve the electricity infrastructure (Special Task Force, 2008). Meanwhile, the government of Bihar has been providing subsidies on diesel to reduce the cost of cultivation and pull the economics in favor of farmers. These enabling policy environment can help to increase the electricity supply to agriculture and by bringing down the irrigation cost can lead to increase in irrigation intensity in near future.

## 4 Conclusion

Sustainable rice intensification in the EIGP will only be achieved at scale if the common practice of cultivating long-duration transplanted rice under rainfed conditions is improved. Timely rice establishment is needed to increase the total system productivity in the region. Timely rice establishment ensures the sowing of next crop during optimum sowing window to realize the maximum yield potential. At present, use of older rice seedlings, water stress, and cold damage cause significant yield losses in most seasons, resulting in very low median yields (simulated at 1.6 t ha^−1^) and high interannual variability (SD 2.1 t ha^−1^). In this study, we applied the APSIM modelling framework to conduct an *ex ante* evaluation of the production benefit of different agronomic interventions to increase yield and yield stability across years for conditions in Central Bihar, India; implications for water productivity were also assessed. Interventions considered include: cultivar length, time of establishment, and supplemental irrigation. These interventions have also been tested in the field by the CSISA project and other initiatives and are judged to have scaling potential with reasonably high levels of farmer acceptance in our target region.

Simulations suggest that single interventions can double yields over prevailing practices as stand-alone innovations, while significantly reducing inter-annual production variability in the case of supplemental irrigation. Additional gains are achievable when interventions are layered: supplemental irrigation paired with medium-duration hybrids increased median rice yields to 4.6 t ha^−1^ with interannual variability reduced by half. In improved systems where irrigation is used to transplant the crop, timely establishment is essential and also influenced by cultivar choice: high and stable yields are achievable through mid-August transplanting if medium-duration hybrids are planted, gaining more than a week’s advantage in operational flexibility in contrast to long-duration cultivars. Transplanting appropriate aged seedlings at the onset of the monsoon can double the productivity due to reduced drought risk for a long-duration cultivar as compare to farmers’ practice but yield variability was little changed.

Alternate rice establishment methods like DSR can help in establishing a rice crop earlier than a conventional transplanted crop. In rainfed DSR systems, the potential pay-offs from single interventions were even higher than for transplanted systems with medium-duration hybrids resulting in a median yield of 4.5 t ha^−1^ against 1.8 t ha^-1^ with long-duration cultivars. For irrigated DSR systems, the optimum sowing window is in early to mid-June which results in higher and stable yields with lower water requirements. Simulation results suggest several risk-reducing improved management pathways that can be selectively matched to farmer risk preferences, investment capabilities, and the diversity of rice production systems in Eastern India. There are, however, significant differences in soil type and rainfall characteristics in the EGP that must be accounted for to confirm our findings beyond the central region of Bihar.
